# Circulating Tumor Cells, Enumeration and Beyond

**DOI:** 10.3390/cancers2021236

**Published:** 2010-06-09

**Authors:** Jian-Mei Hou, Matthew Krebs, Tim Ward, Karen Morris, Robert Sloane, Fiona Blackhall, Caroline Dive

**Affiliations:** 1 Clinical and Experimental Pharmacology Group, Paterson Institute for Cancer Research, Manchester M20 4BX, UK; E-Mails: JMHou@picr.man.ac.uk (J.-M.H.); MKrebs@picr.man.ac.uk (M.K.); 2 School of Cancer and Enabling Sciences, University of Manchester, Manchester Cancer Research Centre, Manchester Academic Health Sciences Centre, Manchester M20 4BX, UK; E-Mail: Fiona.Blackhall@christie.nhs.uk; 3 Christie Hospital Foundation NHS Trust, Manchester M20 4BX, UK

**Keywords:** circulating tumor cells (CTC), circulating tumor micro-emboli, CTC technology, CTC enumeration, CTC molecular characterization, CTC biomarkers

## Abstract

The detection and enumeration of circulating tumor cells (CTCs) has shown significant clinical utility with respect to prognosis in breast, colorectal and prostate cancers. Emerging studies show that CTCs can provide pharmacodynamic information to aid therapy decision making. CTCs as a ‘virtual and real-time biopsy’ have clear potential to facilitate exploration of tumor biology, and in particular, the process of metastasis. The challenge of profiling CTC molecular characteristics and generating CTC signatures using current technologies is that they enrich rather than purify CTCs from whole blood; we face the problem of looking for the proverbial ‘needle in the haystack’. This review summarizes the current methods for CTC detection and enumeration, focuses on molecular characterization of CTCs, unveils some aspects of CTC heterogeneity, describes attempts to purify CTCs and scans the horizon for approaches leading to comprehensive dissection of CTC biology.

## 1. Introduction

Metastatic disease is responsible for over 90% of cancer related deaths [1]. The presence of viable circulating tumor cells (CTCs) is a prerequisite condition for establishing distant metastases [2]. In recent years there have been major technological advances, particularly in cytometric approaches, which have allowed evaluation of CTC utility as prognostic, predictive and pharmacodynamic biomarkers, and have paved the way to a realistic prospect of CTC purification, manipulation and molecular characterization to further understanding of the metastatic process. The enumeration of CTCs has current demonstrable, FDA approved, clinical utility with respect to determining the prognosis of patients with metastatic breast, colorectal and prostate cancers [3,4,5,6]. Questions remain on whether existing technologies capture all CTCs in a patient’s blood sample, and the degree of biological heterogeneity in the CTC population is only now being explored. It is likely that a combination of technologies might be required for an accurate evaluation of CTC number. In addition to informing on patient prognosis, CTCs as a ‘virtual and real-time biopsy’ are also now beginning to be exploited to aid drug development. For example, CTCs can provide pharmacodynamic insights (particularly in patients with cancer types where CTCs are relatively numerous), during early clinical trials of novel therapeutics, a context in which the acquisition of invasive tumor biopsies immediately pre and serially post drug treatment is frequently problematic. Indeed, several studies have revealed the pharmacodynamic behavior of CTCs in breast, colorectal, and prostate cancer [3,4,5,6,7,8]. Moreover, drug target expression can be examined using CTCs as a surrogate for tumor biopsies [9,10], and CTC expression of putative or established drug resistance/sensitivity biomarkers is another potential utility. 

However, even in cancer patients with high CTC numbers, such as those with extensive stage Small Cell Lung Cancer (SCLC) (where thousands of CTCs are detected in 7.5 mL blood [11]), the major challenge for CTC researchers is the prevailing difficulty of CTC purification that would allow the molecular characterization of CTCs. CTCs are outnumbered by white blood cells (wbc) in a whole blood sample by a factor of at least 10^6^ and current technologies enrich rather than purify CTCs from whole blood. Thus molecular profiling of CTCs, which might reveal important predictive information and report changes in CTC biology, for example during tumor relapse, is frustrated by wbc signatures that overwhelm those emanating from the CTC minority. This review will outline the current cytometric methods for CTC detection and enumeration, describe with some examples from our laboratory, how additional information with potential use for drug development can be obtained from CTCs, focus on approaches used to purify CTCs to allow their molecular characterization and conclude with our horizon scanning with respect to achieving a comprehensive dissection of CTC biology.

## 2. Cytometric Methods of CTC Detection

Methods of CTC detection are broadly divided into cytometric and nucleic-acid based approaches [12]. Cytometric techniques usually employ immunostaining to identify and characterize CTCs based on the expression of epithelial markers and/or absence of wbc markers. Nucleic-acid based techniques rely on detection of DNA or RNA that are differentially expressed between tumor and blood cells. However, lack of tumor specific probes and an inability to accurately enumerate CTCs using this approach have been and remain limiting. Given the major improvements in cytometric approaches for CTC research in recent years, the implicit advantage of visualizing intact cells for morphological identification of a malignant phenotype and the opportunity for molecular characterization at both cellular and sub-cellular level, cytometric methods are now the most commonly adopted.

The CellSearch^TM^ technology (FDA approved for prognosis in breast, prostate and colorectal cancer) employs a ferrofluid consisting of nanoparticles with a magnetic core surrounded by a polymeric layer coated with antibodies targeting epithelial cell adhesion molecule (EpCAM) [13]. After immunomagnetic capture and enrichment, cells are fixed, permeabilized and labeled with fluorescent probes to identify and enumerate CTCs. The standard probe set is comprised of anti-CK-Phycoerythrin (PE), specific for the intracellular protein cytokeratins 8, 18 and 19 (characteristic of epithelial cells), DAPI to stain the cell nucleus and anti-CD45-Allophycocyanin (APC) allowing negative selection of leukocytes. The CTC enriched sample is dispensed by the CellTracks^TM^ AutoPrep System into a cartridge that is inserted into a MagNest^TM^ cell presentation device, wherein the magnetic field pulls ferrofluid-tagged EpCAM antibody-labeled epithelial cells to the surface of the cartridge. Sample analysis is performed by the CellTracks^TM^ Analyzer II, a four-color automated fluorescence microscope that scans the entire surface of the cartridge, acquires images and displays any event to the user where CK-PE and DAPI fluorescence are co-located. Images are presented in a gallery format for final classification by a trained analyst. Sensitivity is in the order of 1 CTC per 7.5 mL of whole blood and CellSearch^TM^ technology has been demonstrated to be reproducible, sensitive and validated in numerous and increasing numbers of studies [4,13]. However, picking up CTCs in early stage cancer patients remains a challenge with this technology.

Another innovative and exciting micro-fluidic technology that has recently been developed and applied for CTC detection is a micro-fluidic device, the ‘CTC-chip’, capable of efficient and selective separation of viable CTCs from whole blood samples [14,15,16]. The process is mediated by the interaction of target CTCs with antibody (EpCAM)-coated micro-posts under precisely controlled laminar flow conditions. The CTC-chip identified CTCs in the peripheral blood of patients with metastatic lung, prostate, pancreatic, breast and colon cancer, as well as prostate cancer in early-stage. Like CellSearch, the CTC chip identifies CTCs using DAPI and antibodies to cytokeratins and CD45, followed by automated fluorescent image analysis. In addition to the reported high sensitivity, the CTC-chip also demonstrated impressive enrichment, proven by purity ranging from 1% to 80% of CTCs to wbcs (ratio of CK+ cells to CD45+ cells) [15]. This technology platform heralds further evolution in CTC research but, at the time of writing, it awaits mass production, global availability and site to site variation evaluation. 

In contrast to the positive selection strategy for CTC detection, either based on EpCAM and cytokeratin expression profile, or cell size based discrimination (discussed below), Chalmers and colleagues have optimized comprehensively a purely negative enrichment methodology in which only normal blood cells are targeted and removed, thereby allowing rare, non-hematopoietic cells to be enriched. The enrichment process uses a red cell lysis step followed by immunomagnetic labeling, and subsequent depletion, of CD45 positive cells. They reported that the system was able to reduce the number of blood cells in a cancer patient's sample from 4.05 × 10^9^ to 8.04 × 10^3^ cells per mL, and using this approach CTCs were detected from 20 out of 26 head and neck cancer patients. The average number of CTCs detected was 22 per mL of blood with the number ranging from 282 to 1 [17]. Another interesting approach of enriching CTC by depletion of blood cells was modified from a buoyant density centrifugation system by utilizing tetrameric antibody complex which crosslinks unwanted blood cells to multiple red blood cells, forming immunorosettes and thus increasing the density of the unwanted cells, such that they pellet along with the free red blood cells when centrifuged over a buoyant density medium. Schwarzenbach and colleagues adopted this strategy by using the depletion cocktail including antibodies to a wide range of human cell surface antigens and showed that the enriched cells from prostate cancer patients were prostate specific antigen (PSA)-expressing cells by EPISPOT detection method. Furthermore, they demonstrated significant associations of the number of CTCs with the allelic imbalance frequencies at several markers encoding the cytoskeletal protein dematin, the inhibitor of the cyclin-dependent kinase CDKN2/p16 and BRCA1 [18]. Compared with the aforementioned CTC detection methods, the enrichment of CTC by the negative depletion has no bias with respect to CTC definition and provides further flexibility of studying CTCs with any open questions relevant to tumor biology. Alongside the positive selection strategy for CTC detection, this represents a complementary approach for CTC research.

## 3. Size Exclusion Methods of CTC Detection

Isolation by Size of Epithelial Tumor cells (ISET by ScreenCell) allows direct enrichment of epithelial cells using filtration and size exclusion thereby releasing the dependence for detection on the expression of a selected epithelial marker(s) and thus the potential to uncover CTC heterogeneity. Peripheral blood is diluted with ISET buffer, loaded onto polycarbonate membranes with 8µm calibrated pores and processed by gentle filtration under vacuum [19]. This technique is based on the observation that the majority of leukocytes are the smallest cells in the body and that most flow through the pores whereas larger tumor cells are captured on the membrane. The technique minimizes tumor cell loss by avoiding of immuno-labeling with epithelial specific antibodies for capture and can detect one CTC per mL of blood. The CTCs can be directly visualized and subsequently analyzed by a variety of techniques. Given the fact that EpCAM is not expressed in 100% of tumor types (70–80% of variable cancer types [20]) and the likelihood of biological plasticity during metastases such as epithelial to mesenchymal transition (EMT) and its reversion (mesenchymal to epithelial transition (MET) [21,22,23], ISET and other membrane filtration based system [24] present a set of unique but complementary advantages over immuno-labeling based approaches for CTC research. 

## 4. Heterogeneity in CTCs, Unexplored Territory

Most solid cancers are of epithelial origin [25,26]. Using CellSearch technology, (the current cytometric gold standard), the number of epithelial cells in blood from subjects without a diagnosed cancer is very low and almost never exceeds 1 cell per 7.5 mL of blood [25]. Clearly CTCs enumerated using this technology can, in specified contexts, provide clinically useful data that are approved for clinical decision making. However, there is no specific marker known to be uniformly expressed by all cancer types [20]. Furthermore, although the expression profile of epithelial markers such as EpCAM and cytokeratins by CTCs is likely to be modified dynamically during the process of metastasis, there is little direct clinical evidence for this assumption so far. Though not without some controversy, the process of EMT postulated by some as required for metastasis, involves phenotypic changes in a subset of cells within the primary tumor, during which epithelial cells become more motile and invasive through mesenchymal transition, which then via MET revert back to a more epithelial phenotype upon extravasation in host tissue [21,22,23]. It seems probable that this phenomenon of aggressive malignant transition is not an ‘all or nothing’ event, and a process of incomplete EMT has been suggested [27]. Whilst most CTC studies have focused on single tumor cells found within the circulation, evidence for collective migration (groups of cells moving through tissues in co-ordinated cell clusters [28,29] during cancer progression is emerging and might imply that groups of tumor cells should be found in the circulation as tumor microemboli (CTM), and there is some data to support this contention [30]. One therefore should expect that CTCs with metastatic potential may manifest a broad spectrum of phenotypic changes to varying degrees [21,22,23,27,31], which may limit the applicability of immuno-labeling CTC detection approaches if the biology of CTCs is to be unravelled. Nevertheless, most cytometric CTC platforms provide the opportunity to evaluate either diagnostic or therapeutic tumor markers in a pre-selected pool of CTCs. Therefore cytopathological analysis of CTCs could further identify the malignant nature of CTCs as well as providing pharmacodynamic (proof of concept, proof of mechanism) information in the context of targeted therapy [32,33]. 

### 4.1. Cytopathology of CTCs

**Figure 1:**
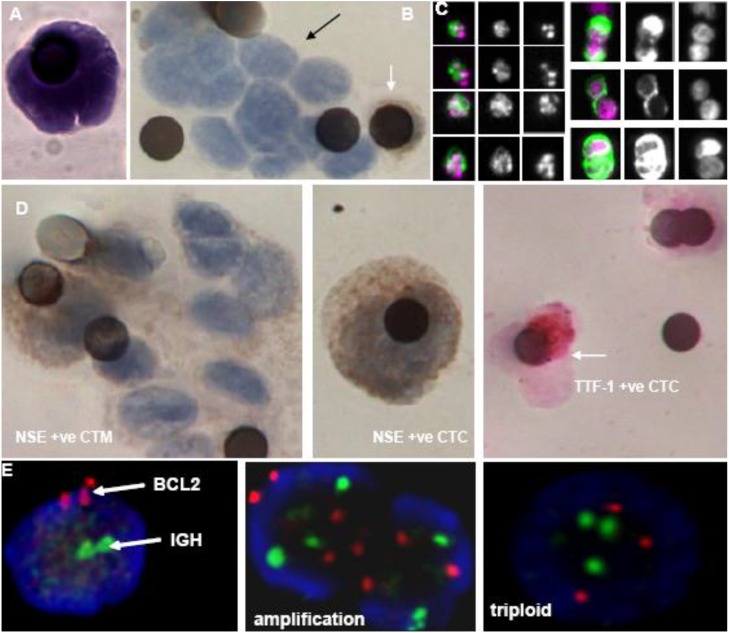
Characterization of CTCs. CTCs isolated from SCLC patients by either the Veridex CellSearch Platform or isolated by ScreenCell ISET size exclusion. **Panel A** shows a single CTC, isolated by ISET and stained by H&E, with high nuclear to cytoplasmic ratio, cell size >8 µm (the pore size of membrane) and irregular nuclear shape. **Panel B** shows a SCLC CTM (black arrow) isolated by ISET alongside a leukocyte positively stained brown for CD45 (white arrow). **Panel C** demonstrates that the DAPI nuclear staining can identify apoptosis (left panel) and nuclear morphology consistent with mitosis (right panel) of CTCs detected by the CellSearch. **Panel D** SCLC associated diagnostic biomarkers, NSE and TTF-1 were immunohistochemically stained with white blood cells used as internal negative control to further confirm the identity of ISET isolated CTM and CTCs **Panel E** shows FISH analysis to detect *bcl-2* genetic abnormalities such as amplification and triploidy from SCLC CTCs. Blood samples were collected from SCLC patients undertaking chemotherapy at the Christie Hospital, Manchester UK according to ethically approved protocols.

Hematoxylin and Eosin stain (H&E) of ISET isolated CTCs showed that cell morphology is retained and CTCs are characterized by large size, high nucleus/cytoplasmic ratio and irregular nuclear shape (Figure 1A). Marrinucci *et al.* conducted several case studies on cytomorphologic analysis of CTCs detected from patients with metastatic colorectal cancer, metastatic breast cancer, or relapsed lung adenocarcinoma using fiber-optic array scanning technology. The detected CTCs were subsequently stained with a Wright-Giemsa stain or Papanicolau stain and compared to the corresponding primary and metastatic tumors. Both the striking similarity and remarkable pleomorphism were found in the CTCs in comparison with the primary tumor tissues or metastatic biopsies [34,35,36]. 

4-6-Diamidino-2-phenylindole (DAPI), the DNA specific dye used in the Veridex CellSearch platform to confirm presence of nucleated cells, also allows discrimination of apoptotic cells with condensed chromatin and nuclear fragmentation [37]. Our SCLC CTC study showed that a positive correlation was observed between apoptotic CTC number (the proportion of morphologically apoptotic CTCs) and circulating levels of caspase cleaved CK18 [11]. In addition to characterizing apoptosis, DAPI staining can also alert to the possibility of mitosis although specific proliferation markers would add clarity (Figure 1C). 

The 4th channel of the CellSearch platform enables further molecular characterization of CTCs and has been used for phenotyping isolated CTCs in several studies. CD56, one of the diagnostic markers for SCLC, was analyzed in CTCs using Alexa Fluor 488-conjugated mouse anti-human CD56 antibody. The antibody concentration was 12 μg/mL, which was optimized by titration, and the integration time for the 4th channel of the CellSearch Analyzer II was configured to 0.4 seconds. The positivity of CD56 in SCLC CTCs was obtained using the research mode of the CellSearch Analyzer II and the results showed that in all blood samples that contained CTCs there were CD56 positive cells, consistent with the CD56 staining profiles from matched tumor biopsies and confirming the dual epithelial and neuroendocrine phenotype and neoplastic origin of CTCs [11]. Similarly, ISET isolated SCLC CTCs have been identified by immunohistochemistry staining which was performed downstream of ISET, for markers of interest such as thyroid transcription factor-1 (TTF-1) and neuroendocrine specific enolase (NSE) (Figure 1D) using standard Envision Kits and the Liquid DAB+ Substrate Chromagen System with white blood cells served as negative staining controls for analysis. 

Circulating tumor micro-emboli (CTM) are observed as multi-cellular aggregates or clusters of epithelial-originated tumor cells present in circulation. The presence of CTM is suggested to be a marker of highly metastatic potential [30]. Our recent data showed that both the CellSearch and the ISET can detect CTM in blood samples from small cell lung cancer (SCLC) patients; ISET had a greater capability of isolating CTM with respect to CTM number and size, which might reflect the reduced requirement for manipulation of the blood sample and/or size constraints of CTM captured by immunomagnetic beads, and/or absent expression of the CellSearch capture and identification antigens, EpCAM and cytokeratins by SCLC CTM (Figure 1B). EMT has been implicated in the process whereby carcinoma cells disseminate from their local environment and metastasize to a secondary site [21,22]. It is postulated that only a small percentage of tumor cells ever undergo a total/complete transition and that it is these cells that are presumably the source of actively metastatic cells [38,39,40,41]. In a pilot study, we examined cellular characteristics of EMT in CTCs/CTM isolated by ISET from lung cancer patients and found that CTCs/CTM demonstrated inter and intra-patient heterogeneity. Amongst the CD45 negative, malignant CTCs/CTM, some but not all cells expressed mesenchymal markers such as Vimentin and N-cadherin. Likewise, some but not all cells retained epithelial characteristics, expressing of E-cadherin and cytokeratins. Our substantial challenge now is to understand the clinical significance of this heterogeneity within tumor cells in the circulation.

### 4.2. Pharmacological Biomarkers Measured in CTCs

As a potential ‘virtual’ biopsy with availability pre- and post-drug treatment, the molecular characteristics of CTCs have been utilized in several studies to serve as predictive biomarkers and/or to provide pharmacodynamic data to inform on targeted therapy. For example, being a predictive biomarker for HER2 targeted therapy, HER2 expression was assessed in advanced breast cancer patients and the results showed that a bi-directional shift in HER2 status between the primary tumor and corresponding CTCs occurred which may help to identify additional patients who could benefit from HER2 targeted therapy [10]. This study implied that there are differences in the molecular profile between the primary tumor and the corresponding CTCs and also reflects the possibilities for clonal selection during treatment. Insulin-like growth factor-IR (IGF-IR) is implicated in proliferation, angiogenesis, apoptosis and carcinogenesis and thus represents a therapeutic approach for malignancy [39]. One study demonstrated that the IGF-IR is frequently expressed on CTCs of patients with metastatic tumors which may suggest the relationship between IGF-IR expression and a more aggressive disease phenotype. Furthermore, the study suggested a potential utility of IGF-IR-positive CTC enumeration as a predictive marker for the identification of patients that could benefit from anti-IGF-IR therapy [9]. Kallergi *et al.* investigated the expression profile of EGFR, HER2, PI3K and Akt in CTCs in breast cancer patients. EGFR and HER2 expression was detected in a subpopulation of CTCs. More importantly, activated EGFR and downstream pathway activation of PI3K/Akt was also observed in CTCs which suggested that EGFR- or HER2-driven PI3K/Akt activation may be involved in the regulation of the malignant and metastatic potential of CTCs. These molecules could serve as therapeutic targets or pharmacodynamic biomarkers for mechanism based therapeutics [42]. Similarly, nuclear proteins have also been explored as PD biomarkers in CTCs. Phosphorylated H2AX (γH2AX) reports DNA double-strand break damage; and nuclear γH2AX levels in patient CTCs following chemotherapeutic treatment were a sensitive pharmacodynamic biomarker that potentially, can enable longitudinal monitoring of a drug-target response [43]. 

### 4.3. Characterization of Cytogenetic Abnormalities of CTCs

Disease staging and personalized cancer therapy depends on molecular information derived from tumors. A clear example of this was the large randomized trial that demonstrated the presence of the *EGFR* gene mutation in a tumor as a strong predictor of a better outcome for EGFR inhibitor treatment. Equally important findings showed that patients with wild type EGFR who received first-line EGFR inhibitor treatment had the Hazard Ratio for progression free survival of 2.85 (95% CI 2.05–3.98; P < 0.001) compared with patients received carboplatin-paclitaxel chemotherapy [14]. This paradigm exemplified for EGFR targeted therapy, is becoming the prevalent one in anticancer drug development where stratification of patients is performed according to the molecular characterization of their tumor. The analysis of CTCs for molecular abnormalities offers an alternative, less invasive and real time approach to the interrogation of archival tumor biopsies. After enrichment of CTCs by the CellSearch system, it is now possible to evaluate them cytogenetically using fluorescence *in situ* hybridization (FISH). More recently CTC-chip technology showed that both the classical EGFR activating mutation, and the emergence of the T790M resistance mutation were detected in CTCs [14]. 

K-Ras, the small G-protein acting downstream of EGFR signaling is an essential component of the EGFR signaling cascade. Activating mutations of *K-ras* thus hijack the pathway with loss of EGFR dependence, thus rendering EGFR inhibitors ineffective [44]. Yang *et al.* established a platform-weighted chemi-luminescent membrane array and demonstrated detection of CTCs harboring *K-ras* mutations in the blood of colorectal cancer patients which could potentially aid to identify *K-ras* wild type patients who are more likely to benefit from treatment with EGFR inhibitors [45].

Several studies of CTC cytogenetics have been conducted regarding prostate cancer. Swennenhuis *et al.* developed a method for assessment of chromosome 1, 7, 8, and 17 copy numbers on CTCs from metastatic castration resistant prostate cancer patients and showed that the enriched cells were indeed cancerous and heterogeneity was observed with respect to the aberrancy of the copy number of these chromosomes between patients, as well as between CTCs in the individual patient [46]. Attard and colleagues reported the copy number changes of key genes like *MYC*, *AR*, *PTEN*, as well as *TMPRSS2/ER* gene rearrangements on prostate cancer CTCs. The results confirmed that CTCs isolated from castration-resistant prostate cancer (CRPC) patients are malignant in origin and indicated that hormone-regulated expression of ERG persists in CRPC [47]. Similar findings from another study showed that high-level chromosomal amplification of *AR* and relative gain of *MYC* were respectively detected in 38% and 56% of CTC samples collected from castration-resistant metastatic prostate cancer patients. These data indicated that androgen signaling continues to play an important role in late-stage prostate cancer [48]. 

We have also developed FISH analysis of CTC-enriched samples downstream of the CellSearch System, where CTCs are pre-FISH imaged before the hybridization and then revisited for the post-FISH imaging. With awareness of Bcl-2 family targeted drugs entering early clinical trials, our feasibility experiment showed that *Bcl-2* genetic abnormalities such as amplification, *Bcl-2/IGH* translocation, and triploidy can be detected in CTCs from SCLC patients (Figure 1E). 

These exemplar studies serve to illustrate the considerable potential for clinical utility of molecular characterization of CTCs as real-time, serial and non-invasive biopsies for evaluation of therapeutic targets as well as the development of novel therapeutics.

## 5. Omics Approaches for Molecular Profiling of CTCs

The molecular characterization of CTCs has garnered renewed attention since the revolutionary technology improvements made for the enumeration of CTCs described above. Cytopathological and cytogenetic analysis of CTCs have been providing information with respect to diagnosis, prognosis and therapeutic surveillance, where research strategies are mainly hypothesis-driven and the information acquired in a candidate approach. In the ‘omics era’, global analysis and discovery-based CTC research could potentially, provide comprehensive information to facilitate our understanding of tumor biology and in particular the process of metastasis. 

The application of genomics to CTC research, though challenging, may lead to discoveries including the identification of metastasis gene signatures, discovery and dissection of tumor initiating ‘stem-like’ cells and uncover mechanisms of chemoresistance and tumor relapse. With the next generation technologies, it is theoretically possible that a CTC sample could be ‘deep sequenced’ in the near future. However, there has been little data reported so far, a fact that reflects the size of this challenge. Global mRNA transcript expression profiling of CTCs has been explored. Using a set of genes with no or minor expression by leukocytes, Sieuwerts and colleagues performed quantitative gene expression profiling on as little as one CTC in the presence of relatively large number of white blood cells [49]. The method has been optimized to perform mRNA expression analysis of up to 96 genes. Smirnov and colleagues generated global gene expression profiles for CTCs using a method that compared the RNA extracted from the CTC-enriched fraction of a peripheral blood sample with the RNA originated from the corresponding CTC-depleted fraction [50]. However, the further application of this strategy was limited by the use of enriched, not purified CTCs, and the use of leukocytes, but not normal tissue or primary tumor, as matched controls. 

There has been rapid progress in onco-proteomics recently that enables quantitative analysis of the protein expression patterns and protein-protein interactions to dissect the complex tumor biology. Characterization of the CTC proteome potentially provides an opportunity to facilitate our understanding of metastasis as well as the discovery of potential biomarkers. Early steps towards proteomic profiling of CTCs have been taken; Wang and colleagues described a sensitive shotgun proteome analysis method based on the use of a surfactant for cell lysis and nano-LC quadrupole time-of-flight MS for peptide sequencing for analyzing the proteomes of 500 to 5000 MCF-7 breast cancer cells, which were spiked into human blood and then recovered by immunostaining of antibodies and flow cytometry cell sorting. The resultant proteome profiles were found to be similar to those of the original MCF-7 cells [51]. However, at the time of writing, proteomic profiles from clinical CTC samples have not been reported. 

The current challenge of applying ‘omics’ technologies to CTC research is that cytometric-based technologies enrich rather than purify CTCs from whole blood and we still face the ‘needle in the haystack’ problem. Consequently, the further purification of CTCs enriched by current technologies is a pressing objective. 

## 6. Purification of CTCs

**Figure 2:**
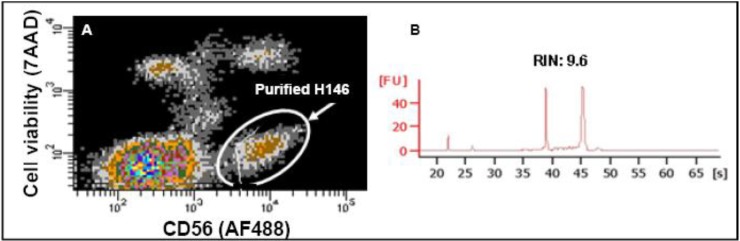
Feasibility evaluation of Tandem CellSearch-FACS for CTC purification. Spiked SCLC H146 cells were processed by the CellSearch system using CellSearch Profile kit. **Panel A** The recovered cells were stained with Alexa Fluor 488 conjugated CD56 antibody and 7-AAD before the sorting of viable CD56 positive cells by FACS. **Panel B** A quality check on extracted RNA from FACS sorted cells in panel A showed good quality of RNA with high RNA integrity number (RIN).

Our own efforts to purify CTCs are ongoing, for example, we are evaluating the feasibility of a tandem CellSearch-FACS (Fluorescence activated cell sorting system) approach. Cultured H146 SCLC cells were spiked into whole blood, which was previously collected into EDTA tube but not CellSave tube, and then the spiked samples were processed by the CellSearch system (using the CellSearch Profile kit in which there is no fixative and staining reagents, with only CTC-capturing anti-EpCAM-coating ferrofluids provided) so as to enrich spiked unstained tumor cells. To overcome the contamination of wbc, recovered H146 cells were subsequently stained with AlexaFluor 488 conjugated anti-CD56 antibody (a neuroendocrine marker) and with 7-AAD to assess cell viability. Sorting of viable (7-AAD negative) and CD56 positive cells by FACS was optimized and the RNA extracted from the sorted cells was of good enough quality for gene expression profile analysis (Figure 2), which gives us some confidence regarding the challenge of microarray analysis of CTCs. Another approach of obtaining purified CTCs for molecular analysis is to use laser capture microdissection (LCM) to obtain CTCs/CTM for example captured on filters by size exclusion. Due to the small sample size, the extracted RNA from purified CTCs/CTM was subjected to whole transcriptome amplification and the yielded cDNA was suitable for global gene expression analysis.

Another avenue for CTC research is *ex vivo* culture. However, considerable method development is required for *ex vivo* expansion of CTCs immediately after their isolation. If viable CTCs/CTM can be isolated (by, for example, size exclusion techniques), and can be labeled, their behavior after injection into nude mice might be revealing. Kojima and colleagues developed a biological imaging system for detecting viable human CTCs using a telomerase-specific replication-selective adenovirus expressing GFP (OBP-401). The detection method involved a three-step procedure, including the lysis of red blood cells, the subsequent addition of OBP-401 to the cell pellets, and an automated scan using fluorescence microscopy. This GFP-expressing virus-based method enables relatively precise enumeration of CTCs and more importantly the simple and potentially efficient way of isolating viable CTCs. However, the impact of the manipulation of CTCs with the adenovirus infection on the molecular profile of CTCs needs to be comprehensively addressed [52]. 

## 7. Horizon Scanning

With continuous improvement to CTCs technologies, CTC research is no longer limited to CTC enumeration and an era of molecular characterization of CTCs has begun. It is intuitive that viable CTCs, as an intermediate both temporally and spatially between primary tumor and metastasis, possess the unique advantages for researchers to study the tumor biology, especially the process of metastasis. However the real significance of CTCs in tumor biology can only be revealed by well designed, properly matched, studies adopting cutting edge techniques such as comparative genomic hybridization (CGH)/array CGH, deep-sequencing, microarray-based expression profile analysis, proteomic expression profile analysis. Some outstanding questions for CTCs can then be specifically addressed: examples include:

(i) will baseline CTC signatures reveal predictive biomarkers for targeted therapy?
(ii) will signatures from CTCs from chemo-sensitive patients *versus* those from chemo-resistant patients inform on mechanisms of drug resistance?
(iii) will longitundinal CTC signatures at diagnosis *versus* disease relapse after initial responses to therapy inform on acquired drug resistance and/or identify a ‘stem like’ tumor cell?
(iv) will comparative signatures from matched primary and secondary tumors and CTCs shed additional light on molecular events important for metastasis?
(v) will CTC signatures aid discovery and development of anti-metastatic drugs?

Ultimately, it seems highly likely given the current momentum of CTC research underpinned by technology breakthroughs that accurate and sensitive detection together with comprehensive investigation of CTC biology is on the horizon. CTC research is set to expand our knowledge of metastasis and assist in the development of more effective therapeutics for cancer patients. 
